# G protein-coupled receptor GPR68 inhibits lymphocyte infiltration and contributes to gender-dependent melanoma growth

**DOI:** 10.3389/fonc.2023.1202750

**Published:** 2023-06-07

**Authors:** Shangmei Ye, Yunfeng Zhu, Dongmei Zhong, Xiaodong Song, Jialin Li, Fang Xiao, Zhilei Huang, Wenjie Zhang, Mingyue Wu, Kangdi Zhang, Fu-li Xiang, Jie Xu

**Affiliations:** ^1^ Institute of Precision Medicine, First Affiliated Hospital of Sun Yat-sen University, Guangzhou, China; ^2^ Department of Critical Care Medicine, First Affiliated Hospital of Sun Yat-sen University, Guangzhou, China

**Keywords:** melanoma, GPR68, gender dependence, infiltration, T cells, NK cells

## Abstract

**Introduction:**

Melanoma is a common and aggressive type of skin cancer with rising incidence rate globally. Gender is one of the determining factors, and overall males have a higher risk of developing melanoma as well as worse prognosis. Emerging evidence show that GPR68, a G protein-coupled receptor that is sensitive to acid and mechanical stimulations for cellular microenvironment, plays an important role in tumor biology. However, whether GPR68 is involved in gender-dependent regulation of tumor growth is unclear.

**Methods:**

We established a syngeneic melanoma model in *Gpr68*-deficient mice and investigated tumor growth in males and females. The GPR68 activation-induced cellular responses of melanocytes, including intracellular calcium dynamics, proliferation and migration were measured. The landscape of tumor-infiltrating immune cells were analyzed by flow cytometry and the expression various cytokines were checked by qRT-PCR.

**Results:**

GPR68 is required for melanoma growth in males but dispensable in females. GPR68 is expressed and functional in B16-F10 melanocytes, but the activity of the receptor does not directly contribute to proliferation and migration of the cells. GPR68 inhibits infiltration of CD45^+^ lymphocytes, CD8^+^ T cells and NK cells in melanoma in male mice, but has no apparent effect in females. Furthermore, GPR68 functionally inhibits the expression of IFNγ in the tumor infiltrating CD8^+^ T cells and NK cells as well as the inflammatory cytokine expression in the spleen in male mice but not in females. Our results show the gender-dependent modulatory effect of GPR68 on tumor-infiltrating immune cells and their tumor-killing capacity.

**Discussion:**

GPR68 is sensor for acid and mechanical stimulations, which are two important factors in the microenvironment associated with tumor growth and metastasis. Our results suggest a prominent role of the receptor molecules in tumor biology in a gender-dependent manner. Since GPCRs are more feasible to develop small molecule drugs compared to transcription factors, our study demonstrates the potential of GPR68 as a novel druggable therapeutic target for melanoma in male patients.

## 1 Introduction

Melanoma is a common type of skin cancer that arises from melanocytes, the pigment-producing cells of neuroectodermal origin. Its global incidence rate is 15–25 per 100,000 individuals and has been increasing worldwide over the last decades (1). Survival rates in patients with melanoma (cumulative of all forms) vary widely. Early treatment is the key and leads to >90% for 5-year survival rate after primary diagnosis, however, the survival rate of patients with late stage/metastasis tumor lowers to ~20%, indicating that melanoma is one of the most aggressive and lethal forms of cancer (1).

Studies have shown that male melanoma patients have a higher incidence and mortality rate than females, especially after age 45 (2). Melanoma treatment outcomes also show gender differences in chemotherapy and immune checkpoint therapy (3, 4). These differences majorly reflect variations in hormonal factors, genetic susceptibility and immune response between men and women (2). The hormonal effects on melanoma tumor cell as well as immune reaction have been considered as promising research avenues to develop new therapeutic targets for personalized medicine. Studies have suggested that estrogen receptor expression on melanoma tumor samples is negatively associated with tumor thickness and invasiveness (5). Estrogen may have a protective role against melanoma development by modulating melanocyte proliferation (6) while androgen enhances melanoma cell proliferation and tumor growth (7). Moreover, it is well-documented that females have higher baseline levels of immune activation (8–10), such as greater number of CD4^+^ T and B cells, higher activation and proliferation of T cells, and more immunoglobin production in response to pathogen and antigen. On the other hand, there are more CD8^+^ T, T_reg_ and NK cells in male. Additionally, the sex hormones also have regulatory roles on immune cells *via* their receptors. The complexity of pathways regulating tumor growth and microenvironment contributing to the gender difference of melanoma remains unclear.

Studies have shown that host microenvironmental GPR68 deficiency reduced growth of melanoma B16-F10 cell tumors in a syngeneic tumor model (11, 12) using male mice. Gpr68^-/-^ male mice showed enhanced CD8^+^ T cell infiltration in the B16-F10 cell tumor (12), suggesting that GPR68 may have a tumor-promoting role in host cells. Although GPR68 is highly expressed in malignant melanoma (13) and skin cutaneous melanoma (14), reduction of Gpr68 mRNA level by shRNA in the B16-F10 cells showed no effects on tumor growth *in vivo* (12). GPR68 was first identified as a proton-sensing G-protein-coupled receptor (GPCR) that responds to extracellular acidity and regulates a variety of cellular functions (15). Our previous study showed that GPR68 is also a sensor of fluid shear stress caused by blood flow and regulates flow-mediated dilation and outward remodeling in arterioles (16). Moreover, GPR68 is also suggested to be a coincidence detector of extracellular matrix stiffness in combination of low pH and mechanical stimulations (17). The presence of acid and mechanical stimulation is considered a defining hallmark of the tumor microenvironment (TME) (11, 12, 18–21), and GPR68 could play a critical role in this setting. GPR68 expression is highly upregulated in many types of cancer (14). Emerging evidence has revealed that GPR68 may play crucial roles in tumor biology, including tumorigenesis, tumor growth, and metastasis (11, 12, 22–25). As gender difference have been observed in melanoma patients, we investigated the effects of GPR68 in tumor growth using syngeneic tumor models in both male and female mice, with the goal to understand the underlying mechanism regulating gender difference from a novel perspective of receptor signaling molecules.

## 2 Results

### 2.1 GPR68 deficiency inhibits melanoma growth in male mice only

**Figure 1:**
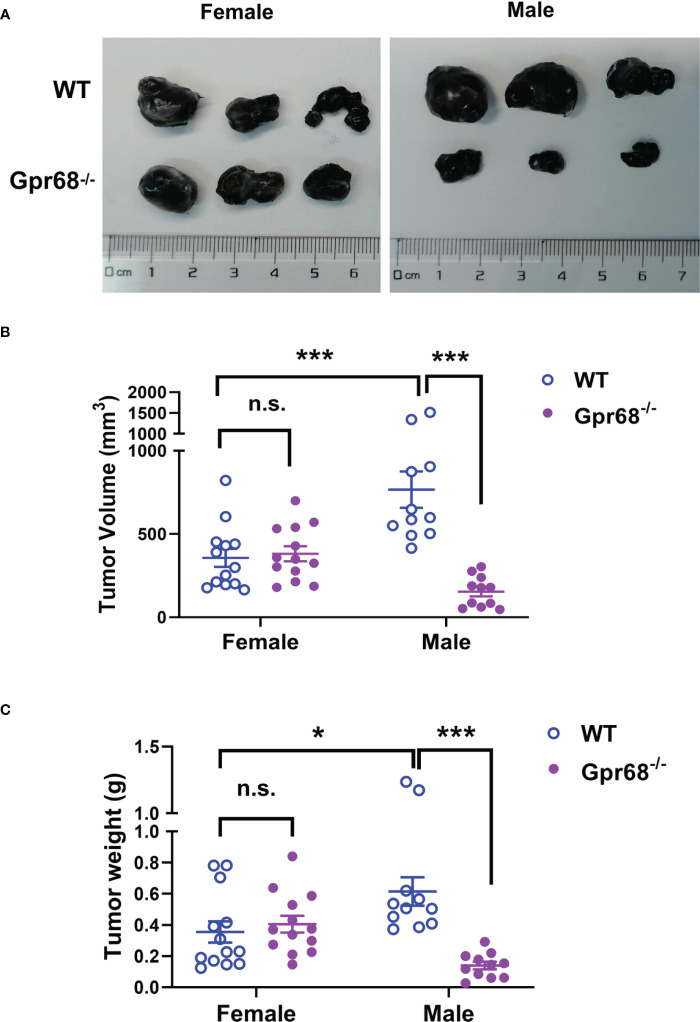
GPR68 deficiency inhibits melanoma growth in male mice but not in females. **(A)** Representative images of melanoma isolated 11d after 1×10^6^ B16-F10 cells were injected into subcutaneously in male and female WT and *Gpr68* ^-/-^ mice. **(B, C)** The size and weight of tumors was measured and volume calculated. Data is from 13 WT females, 13 *Gpr68* ^-/-^ females, 11 WT males and 11 *Gpr68* ^-/-^ males. *p<0.05,***p<0.001, n.s., not significant vs respective control.

Previous studies demonstrated that loss of GPR68 leads to smaller tumor size in male mice in syngeneic melanoma models (11, 12). However, it’s not clear if GPR68 has a similar effect on female mice. We generated a Gpr68 knock-in mouse line (**Supplementary Figure 1**) to investigate if whether there is a gender difference in GPR68’s protumor role. We injected mouse melanoma cell line B16-F10 subcutaneously in WT and Gpr68^-/-^ mice of both sexes and monitored the growth of tumor over time. Consistent with previous studies, we found that the tumor in WT male mice grew to ~800 mm^3^ and ~0.7 g on average at day 11-14, but tumor in Gpr68^-/-^ male mice is significantly smaller (**Figure 1**). In females, however, the size and weight of the tumors were significantly smaller than WT males, albeit to a less extent (**Figure 1**). Interestingly, the tumor growth shows no difference between female WT and Gpr68^-/-^ mice (**Figure 1**). This suggests that GPR68 is critical for regulating melanoma growth *in vivo* and its role is gender dependent.

### 2.2 GPR68 in B16-F10 cells do not affect proliferation and migration

**Figure 2:**
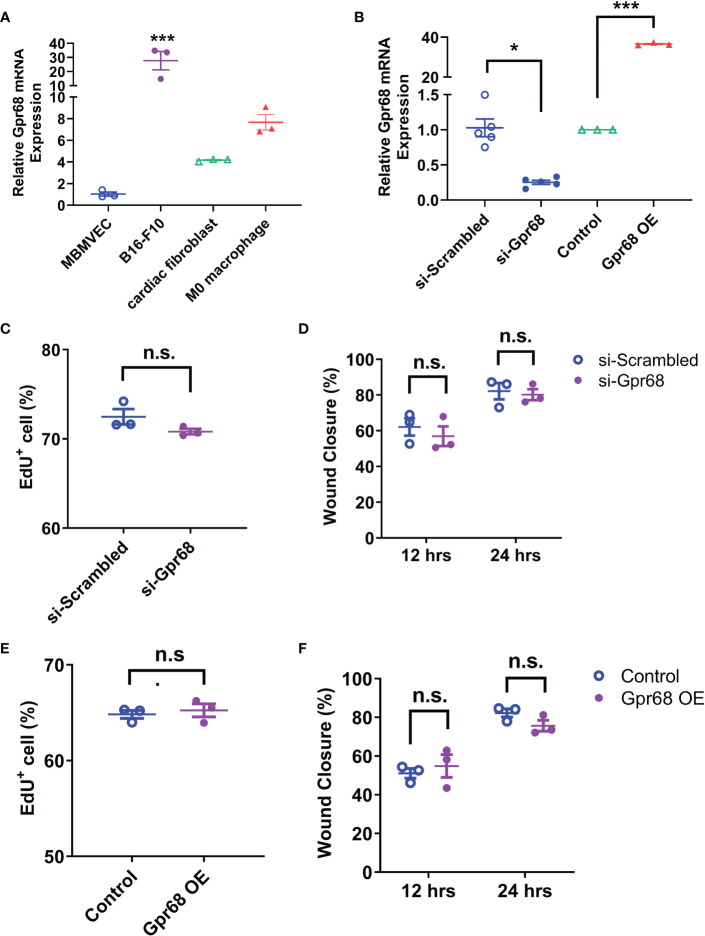
GPR68 does not affect migration and proliferation of B16-F10 cells. **(A)** The relative level of *Gpr68* mRNA is determined by qRT-PCR in mouse brain microvascular endothelial cells (MBMVECs), B16-F10 cells, primary cardiac fibroblasts and primary M0 macrophages. All data is normalized to the mean of MBMVEC. ***p<0.001 vs all other three groups. **(B)** Transfecting B16-F10 cells with siRNA against *Gpr68* reduces its mRNA by ~75%, indicating an effective knockdown. Overexpression of *Gpr68* by infecting B16-F10 cells with lentivirus containing mouse *Gpr68* CDS raises mRNA level by >30-fold. *p<0.05, ***p<0.001 vs respective control groups. **(C)** A wound was created by scratching of a confluent monolayer of B16-F10 cells. Cells transfected with *Gpr68* siRNA close the wound at the similar percentage as control cells at 12h and 24h. **(D)** B16-F10 cells transfected with *Gpr68* siRNA shows same percentage of EdU^+^ cells compared to control cells. **(E)** B16-F10 cells overexpressing *Gpr68* show similar rate of wound closure compared to control cells. **(F)** The percentage of EdU^+^ population is not significantly different in *Gpr68*-overexpressing B16-F10 cells and control cells. In all panels, three separate experiments were conducted. n.s., not significant.

To find out if GPR68 exert its effect on regulation melanoma growth by direct effect on tumor cells, we first sought to determine the effects of GPR68 knockdown (by siRNA against mouse *Gpr68*) and overexpression (OE, by transiently transfecting plasmid containing CDS of *Gpr68*) on melanocyte activities. We measured the endogenous GPR68 mRNA level in B16-F10 cells by qPCR and found that it is highly expressed, compared to brain microvascular endothelial cell line (BMVECs), primary mouse cardiac fibroblast and primary mouse monocyte-derived macrophages (**Figure 2A**). Knockdown of *Gpr68* gene was achieved in B16-F10 by siRNA with efficiency more than 70% (**Figure 2B**). B16-F10 cell proliferation and migration were examined by EdU and wound healing assay. No difference in EdU^+^ cell percentage and wound closure ratio between si-Scrambled and si-Gpr68 group were observed (**Figures 2C, D**). Meanwhile, overexpression of *Gpr68* was achieved by lentiviral infection with a more than 30-fold gene expression increase in B16-F10 (**Figure 2B**). No difference in EdU^+^ cell percentage and wound closure ratio between control and *Gpr68* OE group were observed (**Figures 2E, F**). Taken together, these results suggest that GPR68 activity in B16-F10 melanocytes do not directly contribute to key cellular phenotype related to tumorigenesis.

### 2.3 GPR68 in B16-F10 cells do not respond to β-Estradiol or testosterone stimulation

**Figure 3:**
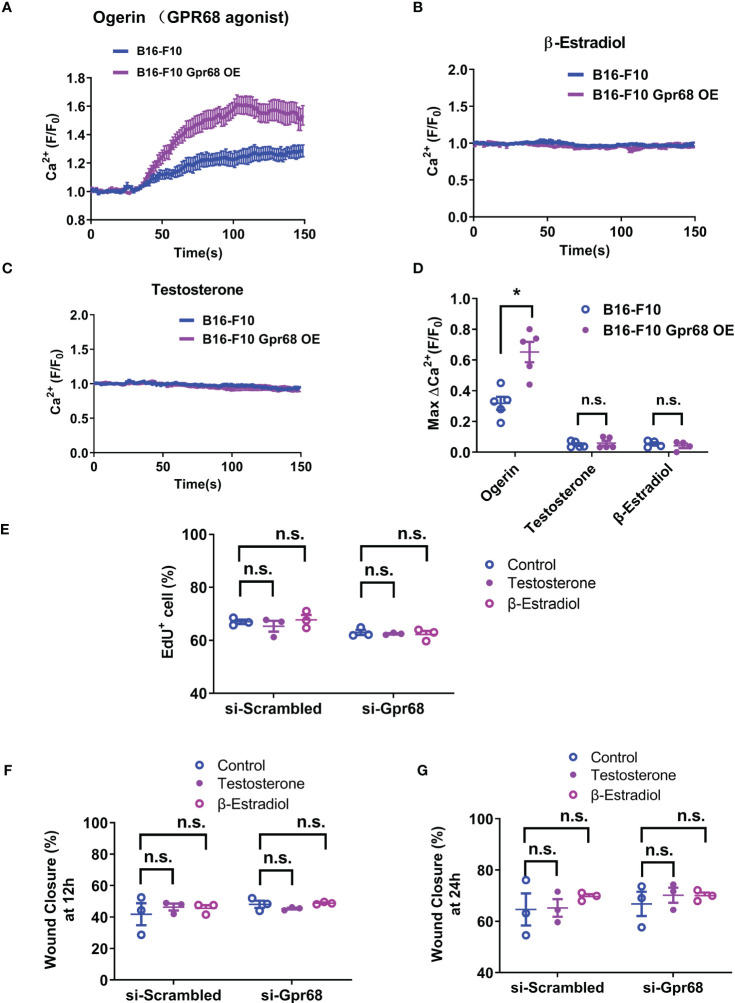
B16-F10 cells respond to GPR68 agonist Ogerin but not to β-Estradiol or Testosterone stimulation. **(A)** Representative response of B16-F10 cells to Ogerin. Cells were loaded with calcium indicator Fluo-8. Background fluorescence was recorded initially and Ogerin was added at 30s (final concentration 50 µM). Traces were the average of fluorescence from ~150 individual cells plotted as mean ± s.e.m. **(B, C)** Representative response of B16-F10 cells to 50 µM of β-Estradiol or 5 µM of testosterone. The respective compounds were added at 20s. Traces were mean ± s.e.m. from ~150 individual cells. **(D)** Quantification calcium imaging experiment. Calcium level changes was shown as fractional increase over baseline and data is from 4~5 trials of ~150 cells each. **(E)** B16-F10 cells transfected with *Gpr68* siRNA with 10nM of β-Estradiol or 100nM of testosterone shows same percentage of EdU^+^ cells compared to control cells. **(F, G)** B16-F10 cells transfected with *Gpr68* siRNA with 10nM of β-Estradiol or 100nM of testosterone show similar rate of wound closure compared to control cells. *p<0.05, n.s., not significant.

To see if sex hormones directly regulate GPR68 activity in the tumor cells, we examined the effect of β-Estradiol and testosterone on GPR68-mediated calcium transients in B16-F10 cells. We used live cell calcium imaging technique to see if downstream second messenger Ca^2+^ can by induced when GPR68 is activated by Ogerin. Ogerin is a specific GPR68 agonist and was shown to be effective in inducing GPR68-dependent physiological effect in *ex vivo* setting (16, 26). We found that 50 µM Ogerin can effectively induce intracellular Ca^2+^ increase in B16-F10 cells (**Figures 3A, D**). We also overexpressed GPR68 in those cells by infecting them with lentivirus containing the cDNA of the gene and observed same response at a significantly higher amplitude (**Figures 3A, D**), supporting the notion that GPR68 is expressed and functional in B16-F10 cells but the exact role remains unknown. Calcium signal was analyzed after treatment of β-Estradiol and testosterone. No calcium signal was observed in both treatment, suggesting that sex hormones do not directly activate GPR68 (**Figures 3B–D**). Furthermore, no difference in EdU^+^ cell percentage and wound closure ratio were observed after treatment of β-Estradiol (10nM) and testosterone (100nM) in si-Scrambled and si-Gpr68 group (**Figures 3E–G**) Taken together, these results exclude the possibility that the gender-dependent differential effect on melanoma growth is due to direct regulation of GPR68 by sex hormones.

### 2.4 GPR68 regulates CD45^+^, CD8^+^ lymphocyte and NK cell infiltration in male background

**Figure 4:**
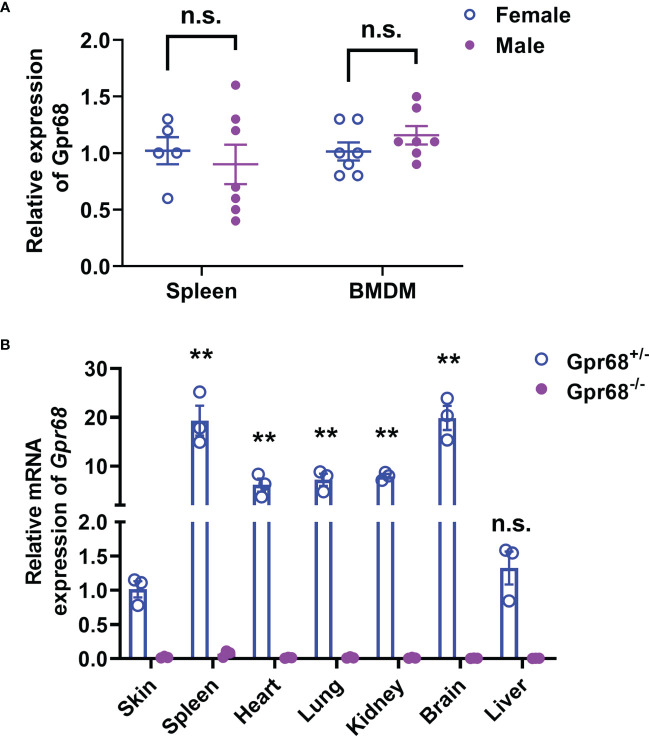
*Gpr68* mRNA level is not significantly different between males and females. **(A)** Relative level of *Gpr68* mRNA in spleen and primary bone marrow-derived macrophage (BMDM) was determined by qRT-PCR. Data is from tissue and cells isolated from 5~7 mice. n.s., not significant. **(B)** Relative *Gpr68* mRNA level from a panel of tissues isolated from *Gpr68* ^+/-^ and *Gpr68* ^-/-^ mice. Expression levels are normalized to the ones from skin of *Gpr68* ^+/-^ mice. n=3 mice from each line. ** p<0.01 vs *Gpr68* ^+/-^ skin group. n.s., not significant.

Since GPR68 expressed in tumor cells have no direct impact on melanocyte migration and proliferation, it is highly plausible that the tumor microenvironment or the immune system has a determining effect on tumor growth *in vivo*. At baseline, no difference of *Gpr68* gene expression in spleen and bone marrow derived macrophages (BMDMs) was observed between male and female mice (**Figure 4A**). Moreover, the expression of *Gpr68* gene was found to be the highest in spleen compared to heart, lung, kidney, and liver (**Figures 4B**) implying its role in immune system. We further analyzed the immune cell population in spleen of WT and Gpr68 KO mice from both genders and found no difference in major immune cell types (**Supplementary Figure 2**).

**Figure 5:**
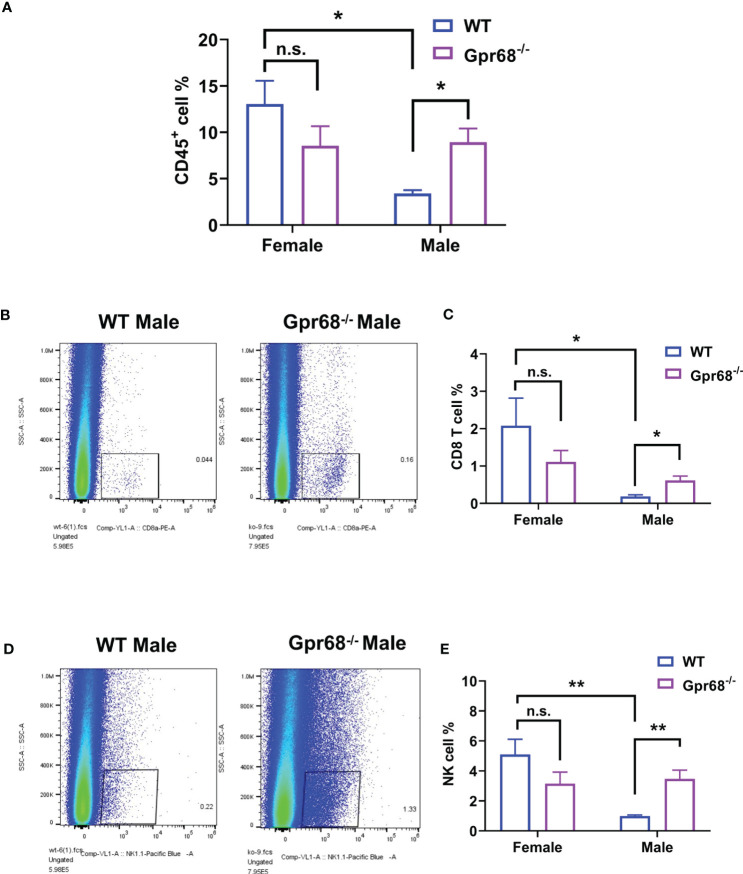
GPR68 deficiency increases percentage of CD45^+^ cell, CD8^+^ T cells and NK cells infiltration in melanoma but not in females. Flow cytometry analysis from cells dissociated from WT and *Gpr68* ^-/-^ tumor tissues, stained with a panel of antibodies to determine immune cell infiltration. We analyzed hematopoietic cells (7AAD- CD45^+^ population, **(A)**, CD8^+^ T cells (CD3e^+^ CD8^+^ population, **(B, C)**, NK cells (CD3e^-^ NK1.1^+^ population, **(D, E)**. n=5 for WT female, 5 for *Gpr68* ^-/-^ female, 4 for WT male and 4 for *Gpr68* ^-/-^ male line. * p<0.05, ** p<0.01, n.s., not significant vs respective control groups.

**Figure 6:**
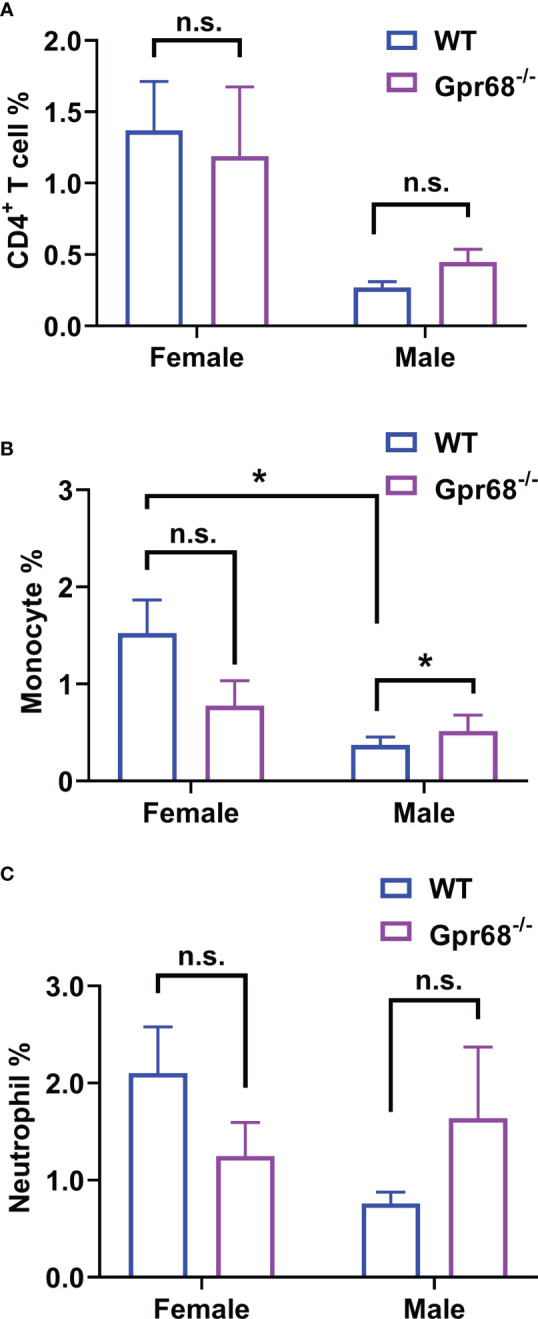
GPR68 deficiency increases monocyte infiltration in tumor but not CD4^+^ T cells and Neutrophils. Cells from tumor tissue was analyzed by flow cytometry. The percentage of CD4^+^ T cells (CD3e^+^ CD4^+^, **(A)**, monocytes (ly6c^+^ CD11b^high^, **(B)** and neutrophils (ly6c^+^ CD11b^mid^, **(C)** were measured. N=5 for WT female, 5 *Gpr68* ^-/-^ female, 4 for WT male and 4 for *Gpr68* ^-/-^ male. * p<0.05, n.s., not significant vs respective control groups.

To gain further cellular mechanistic insight on the differential effect of GPR68 on melanoma growth, we harvested B16-F10 tumor tissues from female and male mice both from WT and Gpr68^-/-^ mice and analyzed the CD45^+^, CD4^+^, CD8^+^ lymphocytes, monocytes, neutrophils, and NK cells infiltrated the tumor and day 11. In females, there’s no difference in the percentage of CD45^+^ lymphocytes isolated from tumor tissue from WT and Gpr68^-/-^ (**Figure 5A**). However, Gpr68^-/-^ male mice has significantly higher infiltrating CD45^+^ lymphocytes, compared to WT male mice, which have infiltration level close to female mice (**Figure 5A**). We observed similar phenotype with CD8^+^ T cells and NK cells inside the tumors, both showed significant higher infiltration in Gpr68^-/-^ males but not in WT males, while in females, both WT and Gpr68^-/-^ have similar levels (**Figures 5B–E**). Meanwhile, other immune cells, including CD4^+^ T cells, monocytes and neutrophiles all had similar presence in tumor tissues among WT and Gpr68^-/-^ mice, in both males and female (**Figures 6A–C**). These results suggest the inhibition of tumor growth observed in male Gpr68^-/-^ compared to WT could be a result of higher CD8^+^ T lymphocyte and NK cell presence, indicating that GPR68 activity in those cells could impact the recruitment and/or migration towards tumor in male background.

### 2.5 GPR68 deficiency increase the expression of IFNγ in tumor infiltrated CD8^+^ T cells and NK cells as well as inflammatory cytokines in spleen in males but not in females

**Figure 7:**
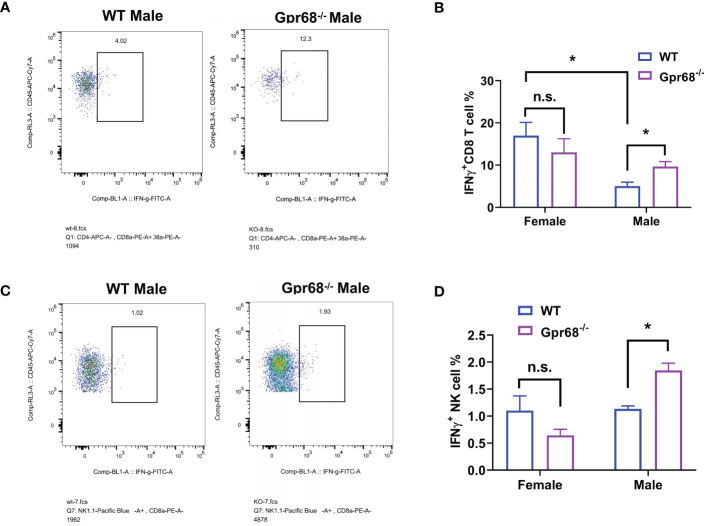
GPR68 deficiency results in significantly higher percentage of IFNγ^+^ CD8^+^ T cells and NK cells in male but not female mice. **(A, B)** Cells from tumor tissues were subjected to flow cytometry to quantify IFNγ^+^ population of CD8^+^ T cells (IFNγ^+^ CD3e^+^ CD8^+^). **(C, D)** Representative flow cytometry plot and quantification of IFNγ^+^ population of NK cells (IFNγ^+^ CD3e^-^ NK1.1^+^) from tumor tissue. n=5 for WT female, 5 *Gpr68* ^-/-^ female, 4 for WT male and 4 for *Gpr68* ^-/-^ male. * p<0.05, n.s., not significant vs respective control groups.

**Figure 8:**
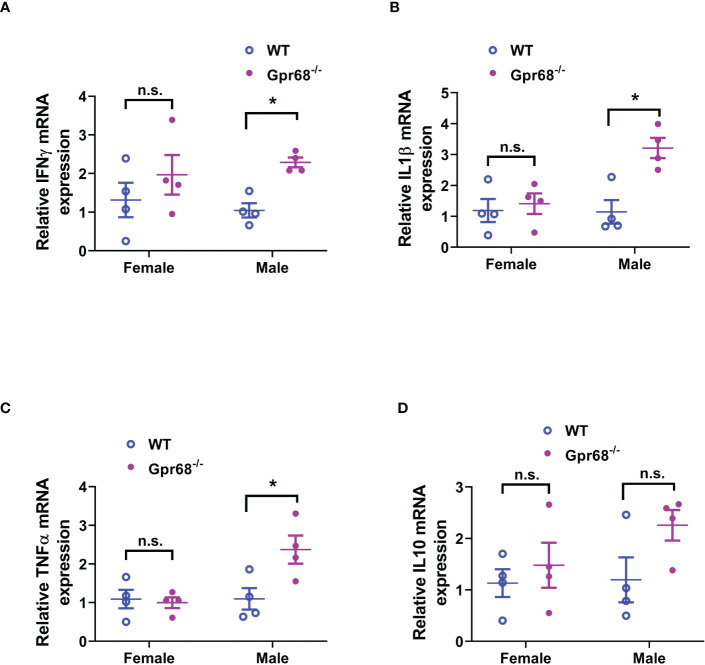
GPR68 deficiency results in significantly higher inflammatory cytokine expression in the spleen of male but not female mice. **(A–D)** Spleens from *in vivo* tumor model mice were subjected to qRT-PCR to quantify the mRNA expression of IFNγ, IL 1β, TNFα and IL 10. n = 4 mice per group. *p<0.05, n.s., not significant.

We’ve shown that female mice, regardless of genotype, have similar CD8^+^ T lymphocyte and NK cell infiltration levels compared to Gpr68^-/-^ male mice (**Figure 5**), yet the tumor growth is not affected. One possibility is that although infiltration is normal with those lymphocytes, their tumor-suppressing activity is somehow inhibited in female mice. To test this hypothesis, we measured the expression of main anti-tumor cytokine IFNγin CD8^+^ T and NK cells by flow cytometry. We found that while the percentage of IFNγ^+^ CD8^+^ T cells is significantly higher in Gpr68^-/-^ male mice compared to WT male mice, in female mice, there’s no difference between the genotypes and both are at a relatively high level (**Figures 7A, B**). Interesting, we discovered that the percentage of IFNγ^+^ NK cells is significantly higher in Gpr68^-/-^ male mice, while it is similar across all other lines (**Figures 7C, D**). This is consistent with tumor volume being low only in Gpr68^-/-^ male mice, suggesting the tumor-suppressing phenotype in male Gpr68^-/-^ mice could be mostly due to higher IFNγ expression in NK cells, but not other cell types. We further analyzed the mRNA expression of proinflammatory cytokines in the spleens from the mouse tumor model. There is a significantly increased IFNγ, IL1β and TNFα in the spleen of Gpr68^-/-^ mice compared to WT in male background but not female (**Figure 8**). Taken together, our results indicate that the differential regulation of tumor growth between males and females are a complex process and likely involves sex-dependent pathways responsible for suppression of CD8^+^ T and NK cells, and sex-independent pathways which might inhibit efficiency of NK cells to kill melanocytes and the spleen immune reaction responding to the tumor.

## 3 Discussion

Gender difference in melanoma incidence and outcome have been proved to be consistent and robust (8, 27). Overall, males have a higher incidence in developing melanoma and higher mortality rate (2, 27). It is well documented that the biological difference in sex hormone and immune system in male and female are responsible for the differences while the mechanisms are not well understood. Here we studied the effect of Gpr68 in a B16-F10 syngeneic melanoma model in both gender and found that tumor growth was repressed in Gpr68^-/-^ male mice not in females. In the repressed tumor tissue in Gpr68^-/-^ male mice, higher percentage of CD8^+^ T and NK cells were observed with a higher level of IFNγ expression. However, neither loss-of-function nor gain-of-function of Gpr68 in B16-F10 melanoma cell line affected cell proliferation and migration. Our data suggest that microenvironmental GPR68 is required for melanoma tumor growth in males but dispensable in females, could be due to inhibition of infiltrating CD8^+^ T cells and NK cells as well as suppression of secretion of IFNγ by those two populations.

GPR68 is highly expressed in many immune cells including T cell, macrophage, NK cell (16). It is originally discovered as an acid sensor (15) which senses the environmental pH change and trigger downstream reaction through second messenger calcium. Our previous study has shown that GPR68 is also a mechanic sensor, which plays a critical role in sensing blood flow and regulating vessel dilation (16). Recently, GPR68 has also been shown to sense ECM stiffness and regulate cell activities accordingly (17). In solid tumor, acid environment and mechanic cues have been considered to associate with tumor growth and metastasis, but the biological mechanisms are not clear. As a proton and mechanic dual sensor, GPR68 have been shown to play crucial roles in tumor biology, including promoting melanoma tumorigenesis in male (11, 12), inducing pancreatic tumor growth *via* fibroblast activation (23), and inhibiting prostate tumor metastasis (22). In the present study, for the first time, Gpr68 was found to associate with gender difference in melanoma growth. Consistent with previous findings, the melanoma tumor size in female WT mice was smaller than male. However, GPR68 deficiency inhibited tumor growth in male mice but had no effects in female.

To illustrate the underlying cellular mechanisms of the sex difference in Gpr68 regulated tumor growth, tumor tissue from both gender and genetic background was harvested for flowcytometry. CD45^+^ cell, CD8^+^ T and NK cells from tumor tissue were found to be lower in WT male compared to female, supporting female advantages in melanoma. Interestingly, Gpr68 deficiency increased CD45^+^ cell, CD8^+^ T, IFNγ expressing CD8^+^ T and NK cells in male mice but had no change between females. Interestingly, the IFNγ expressing NK cells are the only cell population that had no difference between WT male and female but increased in Gpr68 deficient male mice, implying a unique mechanism of Gpr68 in male. It is well demonstrated that CD8 T cells and NK cells are two major tumor killing immune cells. Strategies to enhance CD8 T cell (28) and NK cell (29) tumor infiltration have been shown to decrease tumor growth and accelerate tumor clearance.

Similar to our findings, Cao et al. has reported that host *Gpr68*-deficiency led to increased effector CD8^+^ T cell number and enhanced T cell proliferation, migration as well as IFNγ, TNFα and GramB production (12), which is responsible for the impaired melanoma tumor growth in male mice. Li et al. also showed the anti-tumorigenesis effects in *Gpr68*-deficient mice (gender not mentioned), and claimed the cellular mechanism is through more F4/80 macrophage infiltration (no quantification presented) (11), which is not observed in our study, as well as Cao et al.’s. Although more research is required to clarify the exact roles of the immune cells, it is clear that the anti-tumorigenesis effects of *Gpr68*-deficiency in male mice is likely due to enhanced infiltration of CD45^+^ hematopoietic cells, CD8^+^ T cells and NK cells. This implies a therapeutic potential of GPR68 as a novel target in boosting immune response in male.

GPR68 is also highly expressed in many tumors, such as melanoma (13), pancreatic ductal adenocarcinoma (23), colorectal cancer (30) and medulloblastoma (31). Consistently, we found that *Gpr68* mRNA level is relatively high in B16-F10 cells, and a clear Gpr68 associated calcium signal was observed after Gpr68 activation by its agonist Ogerin. Interestingly, sex hormones did not directly induce GPR68 activation in B16-F10 cells. Moreover, *Gpr68* loss-of-function and gain-of-function in B16-F10 had no effects in cell proliferation and migration, which is consistent with previous report showing *Gpr68* knockdown did not affect melanoma tumor growth *in vivo* (12). These data confirms that the microenvironmental GPR68 is majorly responsible for melanoma tumor growth regulation.

Numerous studies have proved that female immune system is more active compared to male. Clinic observations illustrate that women mount stronger cellular and humoral immunologic responses to foreign and self-antigens and might also be responsible for the gender difference in melanoma development and therapeutic outcome. Our results show that Gpr68 could be a potential target to enhance immunity in tumor microenvironment in males. But the underlying mechanisms remains to be further investigated. One possibility is that the sex hormone receptors (also GPCRs) could directly interact with Gpr68 because interaction between GPCRs could function through same Gs, Gi or Gq pathways. Another possible mechanism could be the interaction of downstream signaling pathway of Gpr68 and sex hormone. Given that it is more feasible to develop small molecule compounds targeting GPCRs, compared to transcription factors, which generally considering undruggable, elucidating the specific pathophysiological mechanisms of GPR68 regulating melanoma growth may provide exciting opportunities for novel drug development against melanoma.

## 4 Methods

### 4.1 Animals

All animals used in this study were maintained under specific pathogen-free conditions and cared for in accordance with National Institutes of Health guidelines. All experiments involving animals were carried out with experimental protocols and procedures reviewed and approved by the Institutional Animal Care and Use Committee of Sun Yat-sen University (SYSU-IACUC-2020-000563).

*Gpr68*-deficient mice were generated on a C57BL/6 background by using CRISPR-Cas9 system to replace the sequences from ATG start codon to TAG stop codon in exon 3 of the mouse *Gpr68* gene with a fluorescent reporter gene encoding a nuclear-localized mRuby protein tagged with Myc-DDK and followed by a rabbit beta-globin polyadenylation signal (SV40 NLS-mRuby-Myc-DDK-rBG pA) (**Supplementary Figure 1**). We obtained homozygous knockout mice (Gpr68^-/-^) by intercrossing heterozygous mice and compared them with wild-type littermates in all experiments. Knockout of *Gpr68* has been confirmed in major organs by qRT-PCR (**Figure 4B**).

### 4.2 Syngeneic melanoma model

To establish a syngeneic tumor-bearing model, 1×10^6^ B16-F10 cells in 100 µL PBS were injected subcutaneously into the right flank of Gpr68^-/-^ and litter mate control mice (8-12 weeks old) (11). Tumor length and width were measured with a caliper to calculate tumor volume using this formula: Volume=1/2 (Length × Width^2^). The mice were sacrificed when they appeared moribund (11–14 days post-injection). The tumors were harvested, and the volume and weight were recorded.

### 4.3 Flow cytometry

Cells from tumor or spleen were isolated by grinding through 70-μm filters and stained with the following fluorochrome-conjugated antibodies: CD45 (30-F11, BioLegend, 103116); 7-AAD (Tonbo Biosciences, 13-6993-T200); CD3 T cells: CD3e (145-2C11, BioLegend, 100305); CD8 T cells: CD8a (53-6.7, BioLegend, 100708); CD4 T cells (GK1.5, BioLegend,100412); neutrophils and monocytes: Ly6C (HK1.4, BioLegend, 115506) and CD11b (M1/70, BioLegend, 101216); natural killer cells: NK1.1 (PK136, BioLegend, 108722), B cells: CD19 (6D5, BioLegend, 115506). For the intracellular IFNγ staining, cells were incubated for 5 h at 37°C in complete RPMI 1640 containing 2 µM Monensin, 50 ng/mL PMA, 1 µg/mL Ionomycin, followed by incubation in BD Perm buffer for 30 minutes at 4°C, washed by BD wash buffer and stained with the antibody IFNγ (XMG1.2, Biolegend, 505806). The stained cells were acquired on the Invitrogen™ Attune™ NxT acoustic flow cytometer system and the data were analyzed with using FlowJo software (BD Bioscience).

### 4.4 Cell culture

The B16-F10 cell line (a gift from Dr. Ce Tang at First Affiliated Hospital of Sun Yat-sen University), was cultured in RPMI-1640 medium (GIBCO, C22400500BT) supplemented with 10% fetal bovine serum (FBS) (Sigma-Aldrich, F8318) and 1% penicillin–streptomycin (GIBCO, 15140122). Cells were maintained in a humidified incubator at 37°C with 5% CO_2_ and the medium was refreshed every two days. The mouse cerebral microvascular endothelial cell line (bEnd.3) was purchased from BIOSPECIES (BIOSPECIES, bEnd.3) and cultured in DMEM (BIOSPECIES, XY-9845) supplemented with 10% FBS and 1% penicillin–streptomycin and containing 1.5% NaHCO_3_. Mouse primary cardiac fibroblasts (mCFs) were isolated from male C57BL/6 mice aged 10-12 weeks. The cells were suspended and seeded into three 6-well plates that were pre-coated with Collagen I (Corning, 354236), with 2 mL of complete DMEM medium (GIBCO, C11995500BT) added to each well. After 3 hours, fresh DMEM medium was added, and the cells were cultured at 37°C with 5% CO_2_. The mCFs were passaged every four days and used up to the third passage. Bone marrow-derived macrophage (BMDM) were harvested from the tibias and femurs of 12-week-old male and female mice and were cultured independently in DMEM (GIBCO, C11995500BT) supplemented with 20% fetal bovine serum, 1% penicillin–streptomycin, and 10ng/mL M-CSF (PeproTech, 315-02-10) on day 0. On day 3, an additional 1mL of the medium containing 20ng/mL M-CSF was added per well. The bone marrow precursors were fully differentiated into macrophages (M0 state) and were ready for experiments by day 7.

### 4.5 Chemicals and solutions

Stocks of chemicals were reconstituted in DMSO (AAT Bioquest, ST038) and stored at -20°C unless stated otherwise. Stock solutions were diluted at least 1:500 in the recording solution to give a final working concentration of 0.02% DMSO. Fluo-8 AM (AAT Bioquest, 21081-5) were dissolved at 1 mM. Pluronic^®^ F-127 (Sigma-Aldrich, P2443) was stored at 10% w/v at room temperature. Ogerin (Cayman, HY-110279) was prepared as a 50 mM stock solution and stored at -80°C. Dihydrotestosterone (Selleck, S4757) were stored as 10 mM stocks. β-estradiol (Sigma-Aldrich, E2758) was stored as a 100 mM stock in absolute ethanol.

### 4.6 Calcium imaging

Cells were seeded into 384-well assay plates (Greiner Bio-One, 788096) and loaded with the Ca^2+^ indicator Fluo-8 AM (AAT Bioquest, 21081-5) in imaging buffer (1x Hanks Balanced Salt Solution with 10 mM HEPES, pH adjusted to 7.4 with NaOH) for 30 minutes at room temperature. Prior to the start of the experiment, a 20-second baseline fluorescence was recorded, then 50 μM Ogerin, 5 μM Dihydro-testosterone or 50 μM estrogen was added and the recording continued for 2 minutes. After the experiment, at least 100 cells were selected for analysis. The intracellular Ca^2+^ concentration was determined by measuring the fluorescence intensity at 488 nm under various conditions. Images were acquired at 1 frame/s. Region of interest representing individual cells were picked and data analyzed in OlyVia software (Olympus) and exported for further analysis (16).

### 4.7 Quantitative reverse transcriptase PCR

Total RNA was extracted using EZ-press RNA Purification Kit (EZBsicience, B0004D), obtained cDNAs with the PrimeScript RT Reagent Kit (Takara, RRO47A), and carried out qRT-PCR using TB Green Premix Ex Taq II (Takara, RB820B) and QuantStudio Real-Time PCR Systems (Thermo Fisher). The PCR conditions were as follows: an initial denaturation step for 30 seconds at 95°C, followed by 40 cycles of amplification at 95°C for 5 s and 60°C for 30 s. To quantify the expression levels, we used 18S as the internal reference gene and calculated the relative expression levels using the 2^-ΔΔCt method (32). qRT-PCR primers: 18S F: 5’-GCCGCTAGAGGTGAAATTCTT-3’, R: 5’-CGTCTTCGAACCTCCGACT-3’; Gpr68 F: 5’-ACCGTGGTCATCTTCCTGGCTT-3’, R: 5’-GCTACACAGTTGAAGCTGGTGAG-3’

### 4.8 siRNA transfection

siRNAs oligos were purchased from Genepharma. To perform siRNA transfection, 50pmol siRNA with 120ul of Opti-MEM and 30ul of Lipofectamine™ RNAiMAX (Life Technologies, 13778) were used in each well of 12-well plate containing 1.5×10^5^ B16-F10 cells. Cells were assayed 48hrs post-transfection. Gpr68 SMARTpool siRNA sequence: 1) 5’-CGAGGAACCUGAAUUGUUA-3’; 2)5’-UAGCUUGAGUCACGUGUAU-3’;3)5’-UAGCUGACCCGGUGCUGUA-3’;4)5’-GGAAUGAGCUGGGAGUGUA-3’.

### 4.9 Lentiviral infection for Gpr68 overexpression

A pLVX-Gpr68 plasmid was reconstructed by modifying the pLVX-8104 vector (kindly provided by Dr Changye Zou) and inserting mouse Gpr68 coding sequence behind the CMV promoter. To produce a virus overexpressing mouse Gpr68, the plasmids were co-transfected with the second-generation packaging vectors into HEK-293T cells using FuGENE^®^ HD Transfection Reagent (Promega, E2311) according to the manufacturer’s recommendation procedures. The medium was changed 16h after transfection, and the supernatant was collected 72h post-transfection. The supernatant was added to cultured B16-F10 cells (1 mL of supernatant, 500 µL of complete RPMI-1640 medium, and 8 µg/mL polybrene per well in a 6-well plate). After 24 hours of infection, we replaced the medium with complete RPMI-1640 medium containing 1 µg/mL puromycin. The recombinant B16-F10 cells were assayed 72h after transfection.

### 4.10 EdU cell proliferation assay

The EdU cell proliferation assay was performed using BeyoClick™ EdU-488 (Beyotime, C0071S) to assess cell proliferation. EdU solution was added to the cell culture medium to a final concentration of 10 μM 2 hours prior to harvesting. Cells were fixed with 4% paraformaldehyde for 15 minutes and washed again with PBS. After permeabilization with 0.3% Triton X-100 and further washing with PBS, Click Additive Solution was added and incubated for 30 minutes at room temperature. Cells were washed with PBS, and Hoechst was added for incubation at room temperature for 10 minutes. Finally, cells were washed three times with PBS and imaged using Olympus BX63F microscope with a CCD camera.

### 4.11 Wound healing assay

A scratch was created in the cell confluent monolayer using a 200 µL pipette tip, and floating cells were washed away with PBS. Serum-free medium was added, and images of the wound area were captured at 0h, 12h, and 24h to track the healing process. The images were analyzed using ImageJ software. Percentage of scratch closure was quantified.

### 4.12 Statistical analysis.

Data were expressed as mean ± SEM. All data sets were analyzed using Graphpad Prism (version 8.0). Unpaired Student t test was used for 2 group comparisons. For data with multiple groups, 2-way ANOVA was performed followed by Bonferroni corrections. A 2-tailed p-value <0.05 was considered to be statistically significant.

## Data availability statement

The raw data supporting the conclusions of this article will be made available by the authors, without undue reservation.

## Ethics statement

The animal study was reviewed and approved by Institutional Animal Care and Use Committee of Sun Yat-sen University.

## Author contributions

SY conducted *in vivo* syngeneic melanoma studies and flow cytometry experiments. YZ carried out *in vitro* siRNA and lentiviral manipulations, characterizations of cellular phenotypes and calcium imaging experiments. DZ verified the deletion efficiency of Gpr68^-/-^ mice. XS, JL and FX conducted RNA expression analysis in tissues and BMDMs. ZH, WZ, MW and KZ provided assistance with experiments. F-lX and JX supervised the project and wrote the paper. SY and YZ contributed equally to this work.

## Supplementary material

Supplementary Figure 1 Genomic sequence details of Gpr68 deficiency mice. **(A)** Gpr68 deficiency is achieved through replacement of endogenous Gpr68 coding sequence by the sequence containing SV40 NLS-mRuby-Myc-DDK-rBG-pA. UTR, Untranslated Regions; CDS, coding sequence. **(B)** Confirmation of genomic DNA sequence replacement by sanger sequencing in Gpr68^-/-^ mice.

Supplementary Figure 2 GPR68 deficiency does not affect percentage of different immune cell population in spleen at baseline. Flow cytometry analysis from cells dissociated from WT and *Gpr68* ^-/-^ spleen tissues, stained with a panel of antibodies to determine immune cell types. No difference was observed between each group. n=3 for female, 4 for males. n.s., not significant vs respective control groups.
