# Supplementary figures and images for: G protein-coupled receptor GPR68 inhibits lymphocyte infiltration and contributes to gender-dependent melanoma growth
Source: Front Oncol. 2023 Jun 7;13:1202750. doi: 10.3389/fonc.2023.1202750 (PMC10282648; doi:10.3389/fonc.2023.1202750)

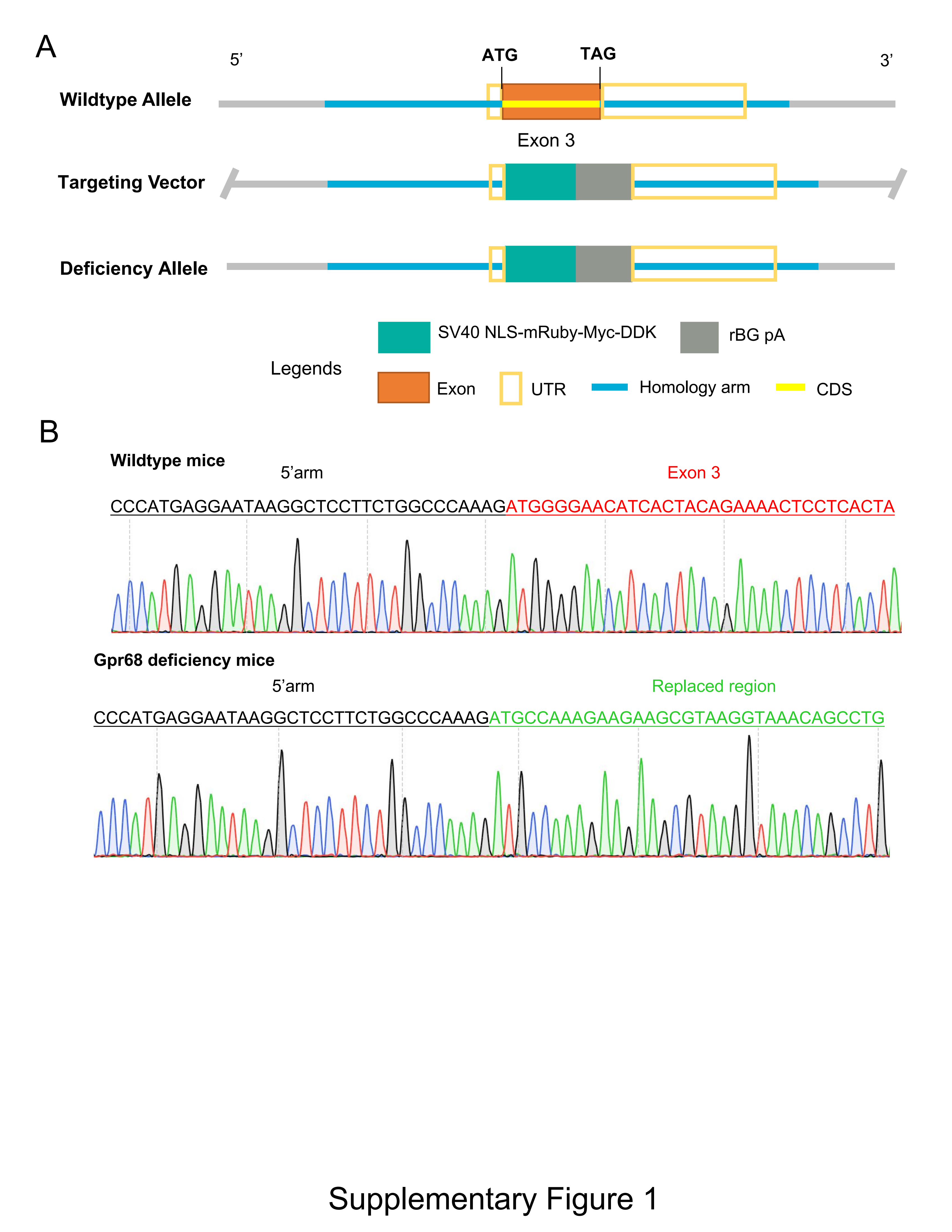

Supplement: Supplementary Figure 1 — Genomic sequence details of Gpr68 deficiency mice. (A) Gpr68 deficiency is achieved through replacement of endogenous Gpr68 coding sequence by the sequence containing SV40 NLS-mRuby-Myc-DDK-rBG-pA. UTR, Untranslated Regions; CDS, coding sequence. (B) Confirmation of genomic DNA sequence replacement by sanger sequencing in Gpr68-/- mice. [file Image_1.tif]

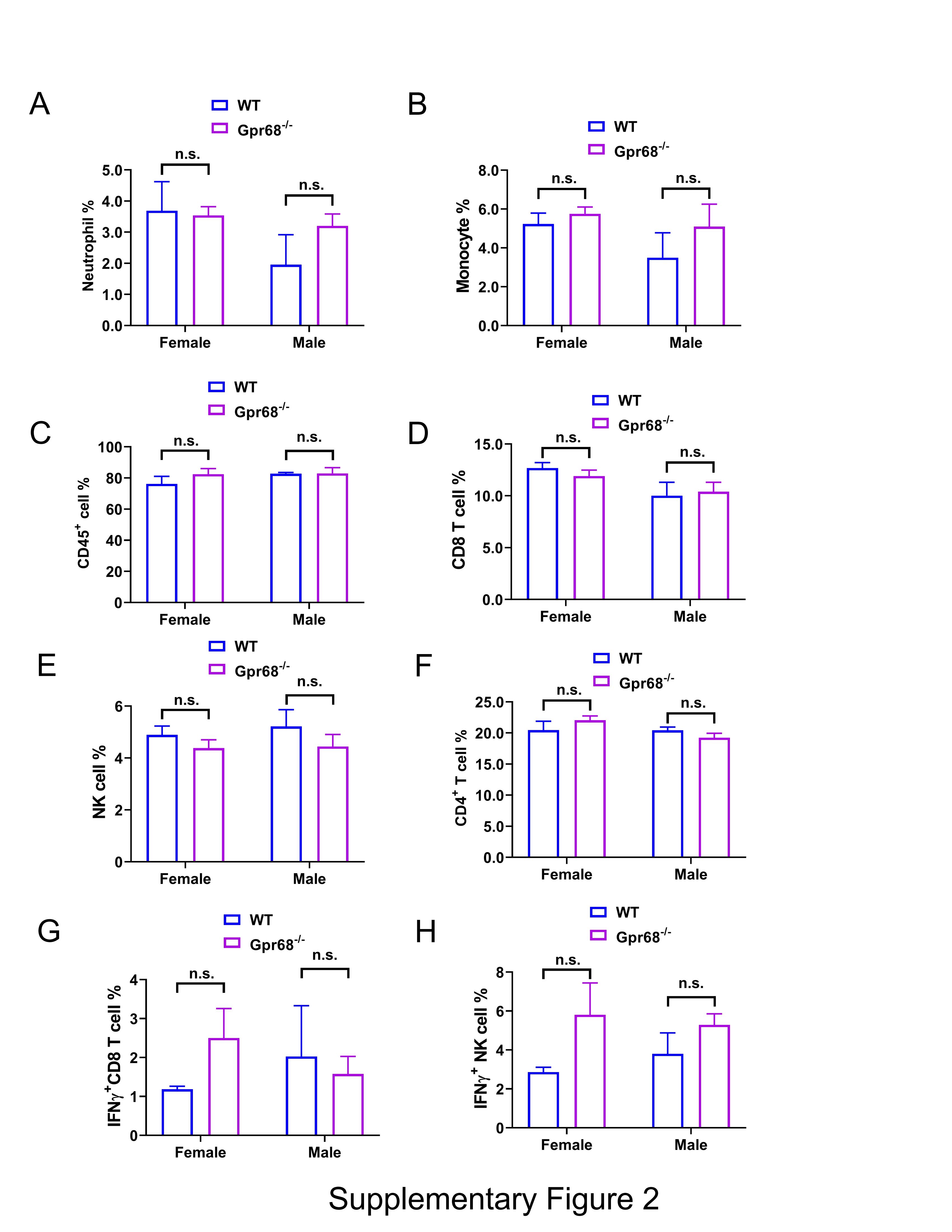

Supplement: Supplementary Figure 2 — GPR68 deficiency does not affect percentage of different immune cell population in spleen at baseline. Flow cytometry analysis from cells dissociated from WT and Gpr68 -/- spleen tissues, stained with a panel of antibodies to determine immune cell types. No difference was observed between each group. n=3 for female, 4 for males. n.s., not significant vs respective control groups. [file Image_2.tif]
